# Connectivity, reproduction number, and mobility interact to determine communities’ epidemiological superspreader potential in a metapopulation network

**DOI:** 10.1371/journal.pcbi.1008674

**Published:** 2021-03-18

**Authors:** Brandon Lieberthal, Allison M. Gardner

**Affiliations:** University of Maine, Orono, Maine, United States of America; Institute for Disease Modeling, UNITED STATES

## Abstract

Disease epidemic outbreaks on human metapopulation networks are often driven by a small number of superspreader nodes, which are primarily responsible for spreading the disease throughout the network. Superspreader nodes typically are characterized either by their locations within the network, by their degree of connectivity and centrality, or by their habitat suitability for the disease, described by their reproduction number (*R*). Here we introduce a model that considers simultaneously the effects of network properties and *R* on superspreaders, as opposed to previous research which considered each factor separately. This type of model is applicable to diseases for which habitat suitability varies by climate or land cover, and for direct transmitted diseases for which population density and mitigation practices influences *R*. We present analytical models that quantify the superspreader capacity of a population node by two measures: probability-dependent superspreader capacity, the expected number of neighboring nodes to which the node in consideration will randomly spread the disease per epidemic generation, and time-dependent superspreader capacity, the rate at which the node spreads the disease to each of its neighbors. We validate our analytical models with a Monte Carlo analysis of repeated stochastic Susceptible-Infected-Recovered (SIR) simulations on randomly generated human population networks, and we use a random forest statistical model to relate superspreader risk to connectivity, *R*, centrality, clustering, and diffusion. We demonstrate that either degree of connectivity or *R* above a certain threshold are sufficient conditions for a node to have a moderate superspreader risk factor, but both are necessary for a node to have a high-risk factor. The statistical model presented in this article can be used to predict the location of superspreader events in future epidemics, and to predict the effectiveness of mitigation strategies that seek to reduce the value of *R*, alter host movements, or both.

## Author summary

Infectious disease outbreaks on human mobility networks often are driven by a small number of superspreader individuals or communities, which are primarily responsible for propagating the disease throughout the network. In this paper, we introduce a model that considers how the properties of the network and spatial variance in disease transmission intensity (i.e., the reproduction number) due to social and ecological conditions interact to influence the occurrence of superspreaders. This type of model is applicable to diseases for which habitat suitability is influenced by climate or land cover, such as vector-borne diseases, and to directly transmitted diseases for which population density and practices to mitigate transmission may vary spatially. We present mathematical models that quantify the superspreader capacity of a population node, based on the extent area of the disease spread attributable to that node and the rate at which the disease spreads. We validate our models with a simulation of epidemic spread across randomly generated networks. The statistical model presented here can be used to predict the location of superspreader events in future epidemics and to predict the effectiveness of mitigation strategies that seek to reduce the disease reproduction rate, alter host movements, or both.

## Introduction

Network spreading phenomena, including epidemic disease spread and information diffusion on social media, tend to be fueled by a small number of individuals in the network. Known as the 20/80 rule or the Pareto principle [1], this pattern is apparent in a variety of infectious disease systems, including the 2003 SARS outbreak in Hong Kong [2], the 2015 MERS outbreak in South Korea [3], and most recently, the COVID-19 pandemic [4]. The concept of superspreaders can be expanded from individuals to include entire communities. In a metapopulation network in which each node represents a city, for example, a few nodes containing highly trafficked airports or other transportation hubs are typically responsible for propagation of the outbreak throughout the network [5]. The identification of these superspreader nodes is an important topic of research in network science and spatial epidemiology, to reduce the area or velocity of disease outbreak spread.

A node’s potential as a superspreader is often estimated based on a variety of network characteristics [6]. Early studies of superspreading dynamics were based on stochastic models, in which each node has a given probability of transmitting the disease to a neighboring node. Therefore, nodes with more neighbors, i.e., higher connectivity, would be expected to spread the disease to a greater portion of the network [7]. More complex models consider not only a node’s degree of connectivity but also the connectivity of its neighbors [8]. Superspreader metrics that consider the structure of the entire network include centrality (i.e., the inverse of the node’s average distance to all other nodes) and k-core values (i.e., the node’s location in the core area of the network) [9], both of which pertain to the potential for a pathogen to disseminate from a given node to the rest of the network. Several studies develop novel definitions of centrality that are particularly well suited to identifying superspreaders because they consider the paths that a pathogen might take to spread through the network [10–13].

None of these network-based approaches to predicting superspreader status consider the common situation that the severity of an epidemic, characterized by either its infection rate or its reproduction number *R*_0_, may vary in space. For instance, for vector-borne diseases, where *R*_0_ is directly related to the habitat suitability for production of the disease vector [14], species distribution models are developed to estimate the probability of presence of vectors based on environmental, climate, and socioeconomic variables [15]. Directly transmitted diseases, such as influenza, are also dependent on spatial factors as their transmission rates may depend on environmental variables such as local temperature and relative humidity [16]. Human factors such as population density and efforts to mitigate disease spread, such as social distancing, quarantines, and sanitation, also affect transmission rates of directly transmitted diseases based on location [17]. Superimposing a metapopulation network, derived from census data and travel routes, on a spatial map of transmission rates to produce a joint metapopulation network/spatial *R*_0_ model is a useful and underexplored approach to visualize and analyze multiple, interacting potential drivers of pathogen spread simultaneously.

Empirical methods to determine superspreader potential typically involve simulating an outbreak originating from a particular node and measuring the extent of disease spread, in terms of the total number of infected nodes, a process which can be computationally intensive for large networks [18]. This study introduces two definitions of superspreader capacity, based on the number of nodes that become infected and the rate of the disease spread originating from a single node, and provides analytical models to predict these based on a node’s connectivity, *R*_0_, and diffusion, along with the properties of its neighbors. We validate these analytical models using a Monte Carlo simulation, involving hundreds of randomized networks superimposed on random *R*_0_ spatial fields. In each simulation, the superspreader capacity of each node is measured, along with several key metrics including degree of connectivity, clustering, centrality, *R*_0_, and diffusion. These data are then used to construct a random forest regression model which predicts the superspreader capacity of any node in a metapopulation network based on its properties [19]. In theory, any real-world metapopulation network, along with a spatial map of *R*_0_ values, can be input to this model to produce a superspreader risk map for a potential future epidemic.

## Methods

### Metapopulation SIR model

The classic SIR (Susceptible-Infected-Recovered) epidemiological model is a mathematical framework to characterize the transmission dynamics of an infectious disease. Individuals from an at-risk population of size *N* are classified among three states (i.e., susceptible; infected; or recovered). Individuals transition from the susceptible to the infected state based upon the transmission rate *β*, and from the infected to the recovered state based upon the recovery rate *μ*. The stochastic SIR model, used in this article, proceeds as follows:

1. The simulation time is incremented by a chosen value d*t*.
2. If at least one individual in the population is infected, a random selection of Susceptible individuals become Infected, as a binomial distribution with probability $1-(1-\frac{\beta *dt}{N}{)}^{I}$, where *I* is the current number of infected individuals. This reflects the assumption that each individual has an independently distributed probability of becoming infected.
3. Simultaneously, a random selection of Infected individuals become Recovered, as a binomial distribution with probability *μ* * d*t*.
4. The simulation repeats, over each time step, until no infected individuals remain.

A metapopulation SIR model is an elaboration of the basic model that considers a network of population centers or nodes, each with its own set of SIR equations, and the rates of population migration between nodes [20]. Individuals migrate from node *i* to node *j* based on a mobility matrix *M*_*ij*_, which is typically calibrated by a diffusion parameter *p* [21]. Here, the processes of transmission, recovery, and migration are simulated stochastically, as described in the Monte Carlo Method section. A node is designated as “infected” when it has at least one infected individual. For the purposes of this study we quantify the spread of the epidemic by the number of infected nodes over time, as we are concerned with measuring the growing area of the epidemic spread.

### Network generation

For our Monte Carlo simulation, we generate a series of random population networks that exhibit properties of scale-free, small-world, and triangulation models [22–24]. This allows us to construct and test models that have a wide range of metrics for connectivity, clustering, and centrality. The algorithm is as follows. A scale-free network with *n* nodes, each representing a homogeneous community, is constructed from *m*_0_ seed nodes to produce a network with degree distribution *P*(*k*) ∝ *k*^−3^ [22]. A forcing algorithm is used to plot this network geographically, scaled so that the average length of any edge is 1. From this, a fraction *b* of all edges are rewired randomly to generate small-world properties (i.e. a short nodal distance between any pair of nodes). Finally, to simulate node clustering, a Delaunay triangulation method is used to connect any nodes within a certain distance threshold *d*. The typical uniformly distributed random ranges of values for each parameter are as follows:

- *n*: 500
- *m*_0_: 1-5
- *b*: 0-0.1
- *d*: 0-1

This algorithm creates networks with an average connectivity degree of 3. The majority of nodes have connectivity less than 10, with hub nodes ranging from 20 to 140. About one third of networks have an average clustering coefficient of nearly 0, the rest are distributed between 0 and 0.5, with most networks between 0.25 and 0.4. The average centrality among networks ranges from 0.14 to 0.26, with a peak at 0.18. This algorithm allows for the rapid generation of human mobility networks that bear a resemblance to real-world case studies [25].

### Monte Carlo method

After a network has been constructed, a random value of diffusion *p* is assigned, ranging from 0.1 to 1, and movement heterogeneity *θ* is assigned as 0.5. A population of 500 * *n* individuals is assigned to the network, such that the population of each node *N* is proportional to *k*^1+*θ*^. All of these individuals are initially classified as Susceptible. The mobility matrix is then defined based on a traffic-dependent model, $M_{ij}=p\frac{{\left(k_{i}k_{j}\right)}^{\theta }}{Ak_{i}^{1+\theta }}$, where *A* is a calibration factor. The recovery rate *μ* is set to 1, and *β* is assigned over a continuous spatial field with an exponential distribution with mean 1.5, to mimic spatial variability in the reproduction rate of seasonal influenza [26]. *R*_0_ fields generated with this method tend to feature a few hot spots with multiple incidences of spatial clustering. Based on this spatial field, an infection rate value *β*_*i*_ is assigned to each node, and a reproduction rate *R*_*i*_ is computed as *β*_*i*_/*μ*.

We introduce 10 infected individuals to one node chosen at random, build a tree data structure with this node at the root, and run a stochastic metapopulation SIR simulation on the network. At each time interval of duration d*t*, the following steps are taken:

1. In each node *i* with at least one Infected individual, a random selection of Susceptible individuals become Infected, as a binomial distribution with probability $1-(1-\frac{\beta_{i}dt}{N_{i}}{)}^{I_{i}}$. A random selection of Infected individuals become Recovered, as a binomial distribution with probability *μ* * d*t*.
2. A random selection of individuals moves from node *i* to each neighboring node *j*, as a binomial distribution with probability $p*dt*\frac{k_{j}^{1+\theta }}{\sum k_{j}^{1+\theta }}$ [27]. Each individual who moves between nodes may be Susceptible, Infected, or Recovered.
3. If neighboring node *j* receives its first infected individual, the current time is recorded, and it is added to the data tree as a subnode under node *i*.

**Fig 1:**
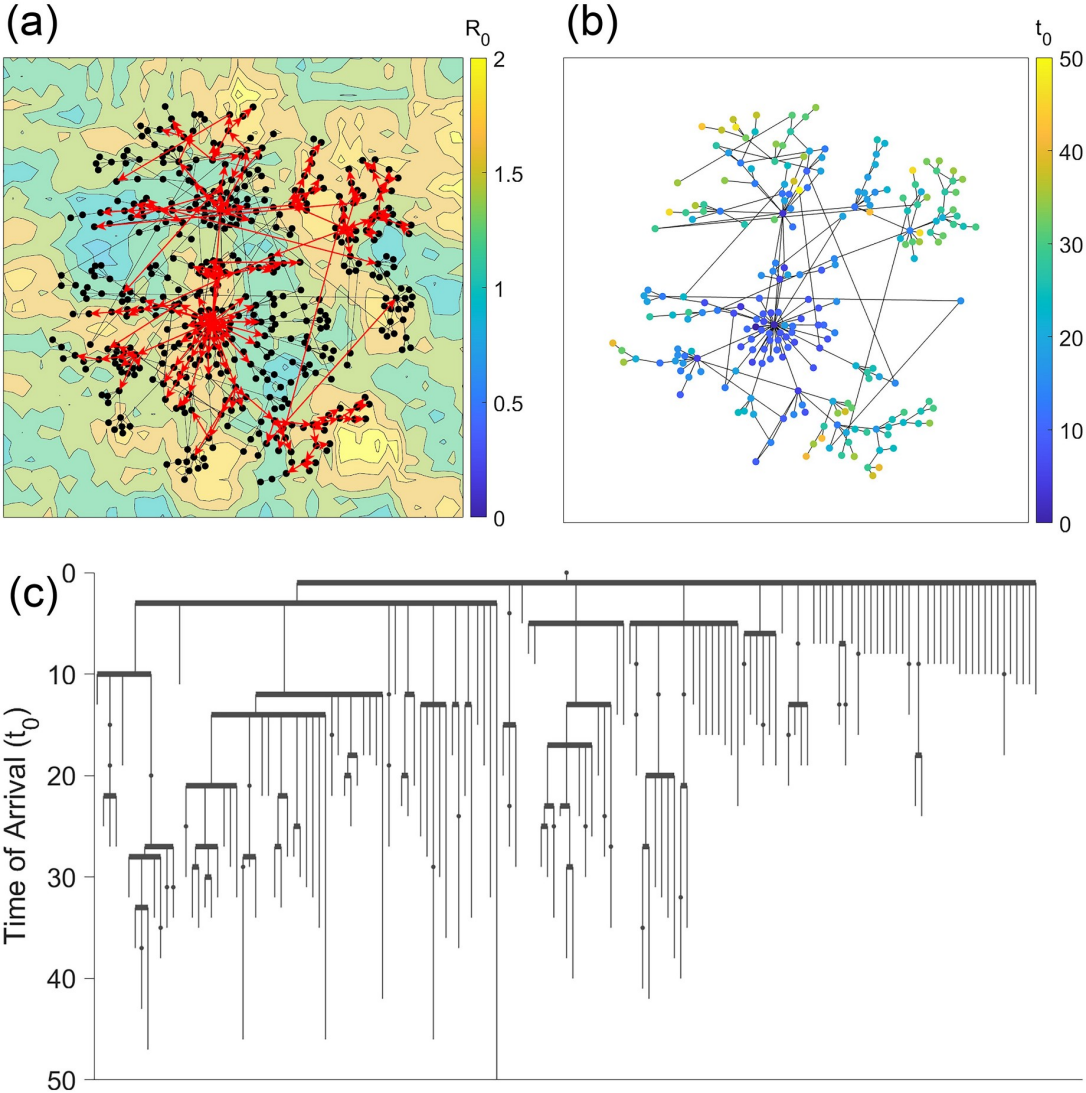
(a) An example of a human network model with 1000 nodes and 1,000,000 individuals. A stochastic metapopulation SIR model was processed on this network, and the red arrows represent the spread of the outbreak. (b) A data tree showing the order in which the outbreak spread throughout the network. Nodes are sorted by the order in which they first spread the infection to a neighboring node, and the y-axis represents the time of arrival of the epidemic.

An example of the data tree is shown in Fig 1. We run the simulation until there are no new infected individuals, typically up to *t* = 750. For each node, we record the epidemic’s time of arrival, peak prevalence, and the infection tree of the network. We repeat this simulation 20 times, each with a different initially infected node, and average our results over these iterations to ensure that the simulation is not biased by the location of the initial outbreak. About 500 networks are processed this way, for a total of about 10,000 metapopulation SIR simulations.

### Superspreader capacity and risk factor

For each simulation, we define two metrics of superspreader capacity for each node:

1. **Probability-dependent superspreader capacity**: the total number of children, including subchildren, of each node on the tree graph. This is analogous to the definition of nodal scope introduced by [28].
2. **Time-dependent superspreader capacity**: the average rate, in nodes per unit time, at which the disease spreads from each node to each of its children.

Each metric is averaged for each node over the 20 simulations run, so that the starting point of the epidemic does not bias our numerical results more than about 20%. Each node is assigned a probability-dependent and time-dependent risk index, ranging from 0 to 1, based on its superspreader capacity among all nodes in its network. We hypothesize that risk indices for probability-dependent and time-dependent superspreader capacity will be strongly correlated, but that time-dependent superspreader risk will be less influenced by the starting point of the epidemic.

We run a random forest model using the randomForest package in R [29] to process a model with risk index as the response (ranging from 0 to 1), and with *R*_*i*_, degree of connectivity, centrality, clustering, and diffusion as predictor variables. The output is a statistical model which takes as input a metapopulation network with an *R*_*i*_ value for each node and outputs a superspreader risk factor index for each node in the network.

### Table of variables

Table 1 gives a list of key variables in our metapopulation model and their definition.

**Table 1:** A list of key variables used in this article and their definitions.

Symbol	Meaning
*n*	number of nodes in the network
*N*	total population of a certain node
*I*(*t*)	number of infected individuals of a certain node
*β*	infection rate of the disease in a certain node
*μ*	recovery rate of the disease
*R*, *R*_0_	reproduction rate of the disease in a certain node
diffusion *p*	the fraction of a nodal population that migrates per unit time
*θ*	heterogeneity of movement of the network
*κ*	the probability that an individual migrates from one specific node to another per unit time
degree of connectivity *k*	the number of adjacent nodes connected to a certain node
centrality	the reciprocal of a node’s average distance to all other nodes in the network
clustering	the fraction of a node’s neighbors that are also connected to each other

## Results

This section is divided into three parts. First, we derive analytically a formula for the probability-dependent superspreader capacity, the expected number of nodes to which a certain node is predicted to spread an epidemic. We apply this formula to various randomly generated networks and discuss the correlation between degree of connectivity, reproduction rate *R*, and superspreader capacity. Next, we derive the time-dependent superspreader capacity, defined as the velocity at which a given node spreads the epidemic to its neighbors, and compare our two definitions of superspreader capacity. Finally, we use a Monte Carlo simulation to develop a Random Forest model relating several network parameters and reproduction rate to a node’s risk of becoming a superspreader site.

### Probability-Dependent superspreader capacity in uncorrelated graphs

To derive a formula for probability-dependent superspreader capacity, consider an uncorrelated network with nodes of varying degree and vector habitat suitability. Within this network, consider a single node *i* with degree *k*_*i*_ and reproduction number *R*_*i*_. This node is adjacent to one node from which the epidemic originated, along with *k* − 1 other uninfected nodes. These *k* − 1 nodes can be decomposed as *k* − 1 = *k*_1_ + *k*_2_ + *k*_3_, where

- *k*_1_ = the number of nodes with *R* ≫ 1
- *k*_2_ = the number of nodes with *R* ≃ 1 (typically between 0.8 and 1.2, say)
- *k*_3_ = the number of nodes with *R* < 1

The node will almost certainly spread the outbreak to its neighbors with *R* ≫ 1, and although infected individuals may travel to neighboring nodes with *R* < 1, it is very unlikely that an outbreak will be able to take root there. Only nodes with *R* ≃ 1 require special consideration.

The probability that node *i* of degree *k*_*i*_ and reproduction number *R*_*i*_ will cause an outbreak in an adjacent node *j* of degree *k*_*j*_ and reproduction number *R*_*j*_ is given by

$$\begin{array}{c} P\left(\text{outbreak}\right)=1-{\left(R_{j}\right)}^{-\lambda_{ij}^{R_{i}}}\;\text{with}\;\lambda_{ij}^{R_{i}}=d_{ij}\frac{\alpha N_{i}}{\mu } \end{array}$$ (1)

The matrix *d*_*ij*_ represents the mobility matrix from node *i* to node *j*, generally a function only of their respective degrees, *N*_*i*_ is the population of node *i*, and *μ* is the recovery rate [21]. The fraction of the nodal population that becomes infected over the course of the outbreak, designated as *α*, is a function of *R*, which can be derived from [30] as

$$\begin{array}{c} \alpha \left(R\right)=\{\begin{array}{cc} 0 & R<1\\ 1+\frac{W(-Re^{-R})}{R} & R\geq 1 \end{array} \end{array}$$ (2)

where *W*() is the product log function. Note that this function is equal to 0 when *R* = 1 and asymptotically increases to 1 as *R* increases to infinity.

We use a traffic-dependent mobility model, which assigns a mobility rate $d_{ij}=p\frac{{\left(k_{i}k_{j}\right)}^{\theta }}{T(k_{i})}$, where $T(k)=\frac{k^{1+\theta }\langle k^{1+\theta }\rangle }{\langle k\rangle }$, and nodal populations are assigned as $N(k)=\frac{k^{1+\theta }}{\langle k^{1+\theta }\rangle }\bar{N}$, where *p* is the diffusion rate, *θ* is the heterogeneity of movement (typically 0.5 or 1), and $\bar{N}$ is the average population among all nodes. This model assumes that a node’s population is proportional to its connectivity, and the rate of mobility between two nodes is proportional to the product of their degrees. The diffusion rate, a fixed value between 0 and 1, represents the average rate of human movement and serves as the constant of proportionality.

If we further assume that *R*_*j*_ ≃ 1, then the outbreak probability can be approximated to first order [21].

$$\begin{array}{c} 1-{\left(R_{j}\right)}^{-\lambda_{ij}^{R_{i}}}≃\lambda_{ij}^{R_{i}}\left(R_{j}-1\right) \end{array}$$ (3)

Substituting the value for λ_*ij*_ from Eq 1 and the value of *d*_*ij*_ from the traffic-dependent mobility model, we derive the outbreak probability:

$$\begin{array}{cc} P(\text{outbreak}) & =\left(p\frac{{\left(k_{i}k_{j}\right)}^{\theta }\langle k_{i}\rangle }{k_{i}^{1+\theta }\langle k_{i}^{1+\theta }\rangle })\frac{k_{i}^{1+\theta }}{\langle k_{i}^{1+\theta }\rangle }\frac{\bar{N}}{\mu }\alpha (R_{i})(R_{j}-1\right)\\ & =p\frac{\langle k_{i}\rangle }{{\langle k_{i}^{1+\theta }\rangle }^{2}}\frac{\bar{N}}{\mu }{\left(k_{i}k_{j}\right)}^{\theta }\alpha (R_{i})(R_{j}-1) \end{array}$$ (4)

The superspreader capacity (SSC) of a node is defined as the expected number of neighbors it will infect in the first generation.

$$\begin{array}{c} \text{Superspreader}\;\text{capacity}\;\text{(SSC)}=k*P(\text{outbreak}) \end{array}$$ (5)

Assuming the node’s neighbors can be divided into *k*_1_ nodes that will definitely be infected (*P*(*outbreak*) = 1), *k*_2_ nodes that have the probability given in Eq (4), and *k*_3_ nodes that cannot support an outbreak (*P*(*outbreak*) = 0), and assuming each outbreak event is independent, the superspreader capacity as defined in Eq 5 can be decomposed as:

$$\begin{array}{cc} SSC_{1} & =k_{1}*1+k_{2}\langle \sum_{k_{j},R_{j}≃1}p\frac{\langle k\rangle }{{\langle k^{1+\theta }\rangle }^{2}}{\left(k_{i}k_{j}\right)}^{\theta }\frac{\bar{N}}{\mu }\alpha (R_{i})(R_{j}-1)\rangle +k_{3}*0\\ SSC_{1} & =k_{1}+k_{2}\langle \sum_{k_{j},R_{j}≃1}p\frac{\langle k\rangle }{{\langle k^{1+\theta }\rangle }^{2}}{\left(k_{i}k_{j}\right)}^{\theta }\frac{\bar{N}}{\mu }\alpha (R_{i})(R_{j}-1)\rangle \\ SSC_{1} & =k_{1}+k_{2}(p\frac{\bar{N}}{\mu }\frac{\langle k\rangle }{{\langle k^{1+\theta }\rangle }^{2}})(k_{i}^{\theta }\alpha (R_{i}))\langle \sum_{k_{j},R_{j}≃1}{\left(k_{j}\right)}^{\theta }(R_{j}-1)\rangle \\ SSC_{1} & =k_{1}+k_{2}(p\frac{\bar{N}}{\mu }\frac{\langle k\rangle \langle k^{\theta }\rangle }{{\langle k^{1+\theta }\rangle }^{2}})(k_{i}^{\theta }\alpha (R_{i}))\langle R_{j}-1\rangle \end{array}$$ (6)

where the term 〈*R*_*j*_ − 1〉 is the mean only over nodes with *R*_*j*_ ≃ 1. A good practice is to consider only nodes for which the quantity $\left[\left(p\frac{\bar{N}}{\mu }\frac{\langle k\rangle \langle k^{\theta }\rangle }{{\langle k^{1+\theta }\rangle }^{2}})(k_{i}^{\theta }\alpha (R_{i}))(R_{j}-1\right)\right]$ is less than 1. This threshold can vary considerably depending on the values of *p*, *μ*, and $\bar{N}$.

**Fig 2:**
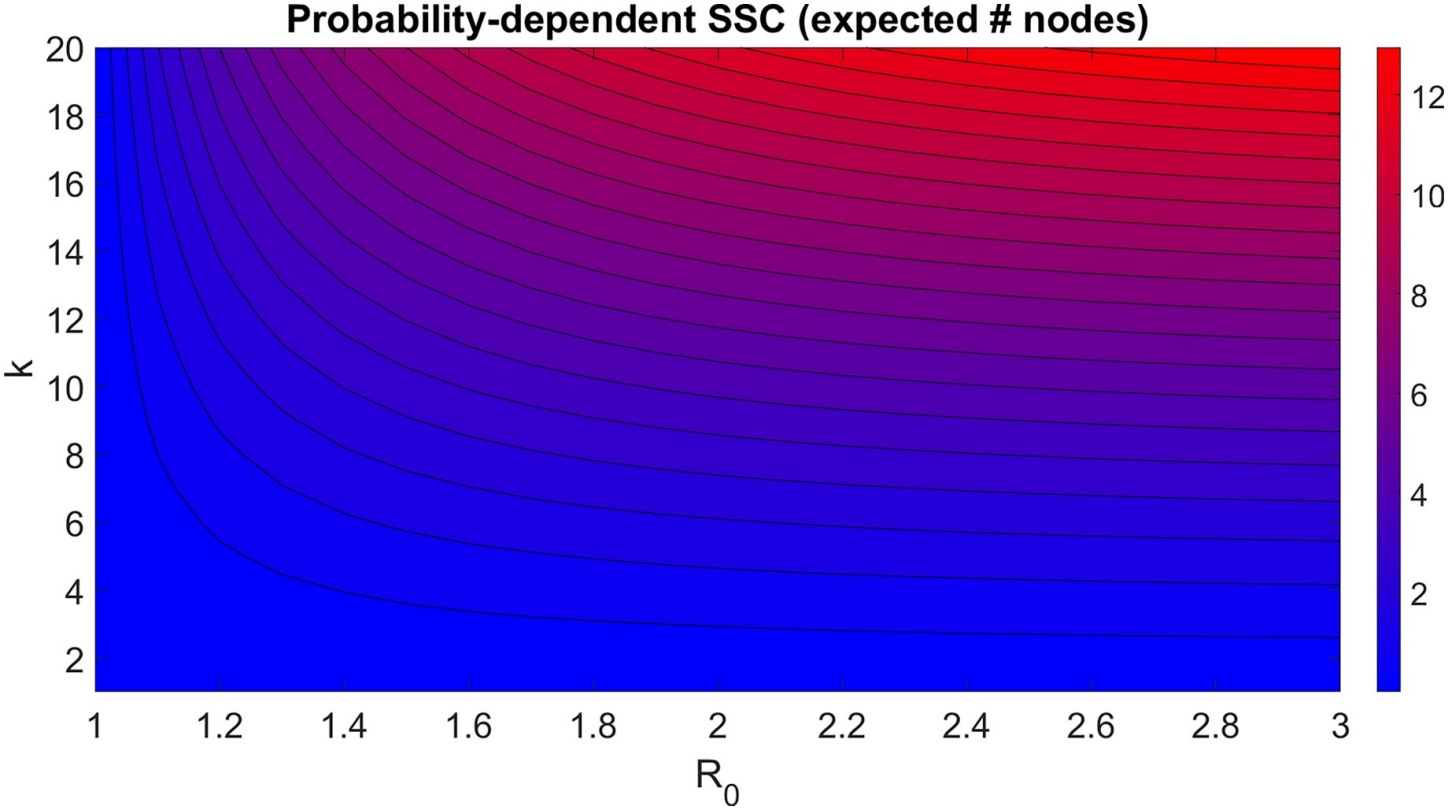
Estimation of probability-dependent superspreader capacity for a node in an uncorrelated network graph, assuming all its neighbors have *R* ≃ 1. In this figure Superspreader capacity is defined as the expected number of neighbors to which the node will spread the outbreak within one generation.

Assuming that *k*_2_ ≃ *k*_*i*_, this implies that the superspreader capacity of the node is proportional to $k_{i}^{1+\theta }*\alpha \left(R_{i}\right)$. A contour plot example of this is shown in Fig 2, with values $p=0.5,\bar{N}=1000,\mu =1,P\left(k\right)\propto k^{-3}$, and 〈*R*_*j*_ − 1〉 = 0.001. The isolines in Fig 2 show nodes with varying values of *k* and *R* that should have the same superspreader capacity. For example, a node with *k* = 14 and *R* = 1.2 has an equivalent superspreader capacity to a node with *k* = 6 and *R* = 2. The effects of *R* on superspreader capacity starts to diminish for *R* > 3, but superspreader capacity increases with *k* indefinitely.

#### Superspreader capacity in classic network models

**Fig 3:**
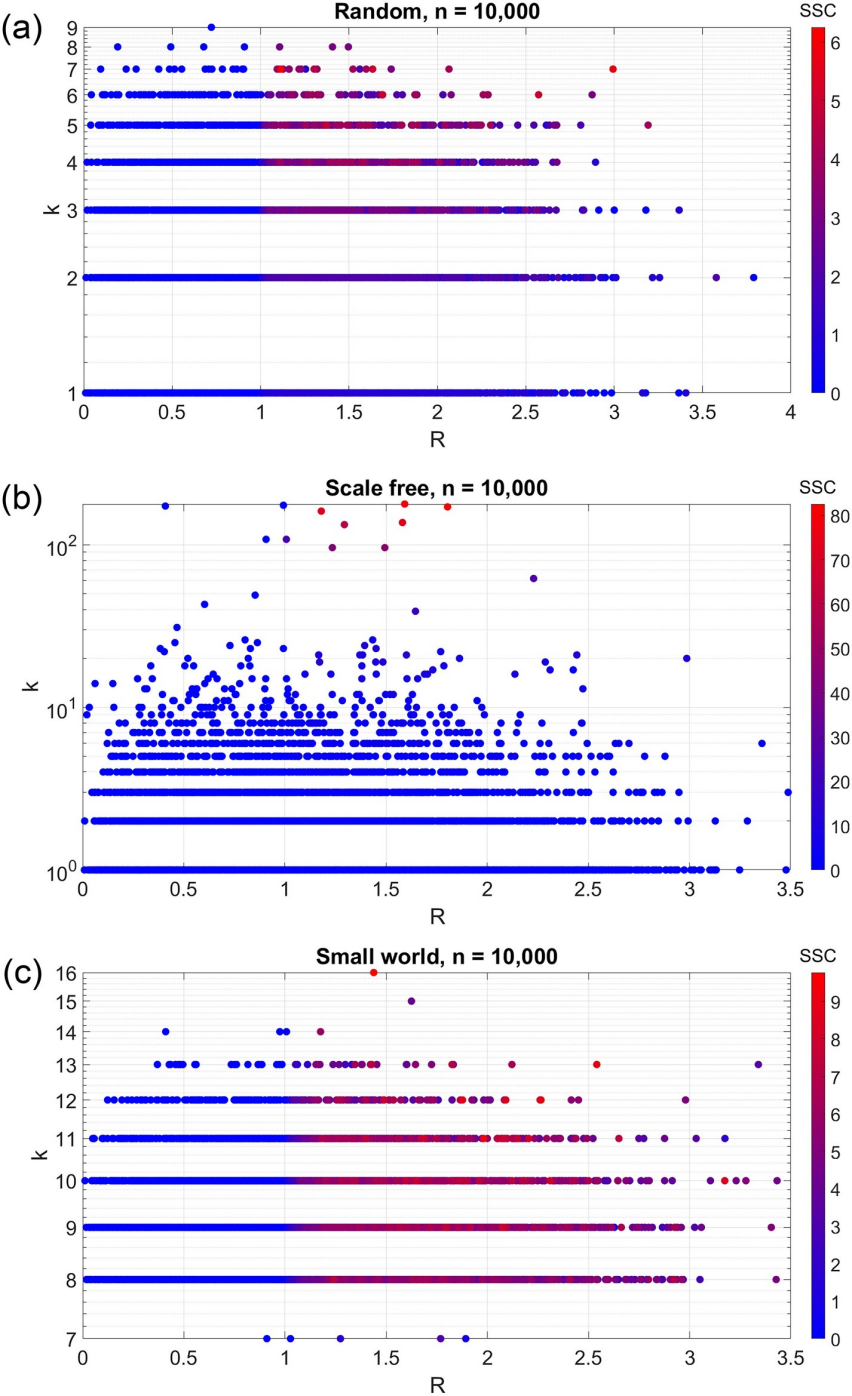
Estimated probability-dependent superspreader capacity in a human population network with 10,000 nodes, with (a) random uncorrelated network, (b) scale free network, and (c) small world network.

The formula given in Eq 4 can be applied directly to a particular metapopulation network to estimate the superspreader risk of each node. To that end, a more precise but less elegant equation for first generation superspreader capacity is found by discarding the assumption that *R* ≃ 1. We substitute Eq 1 and the traffic-dependent mobility model into this definition:

$$\begin{array}{cc} SSC_{1} & =\sum P(\text{outbreak})\\ SSC_{1} & =\sum_{k_{j},R_{j}>1}[1-{\left(R_{j}\right)}^{-\lambda_{k_{i}k_{j}}^{R_{i}}}]\\ SSC_{1} & =\sum_{k_{j},R_{j}>1}[1-{\left(R_{j}\right)}^{-p\frac{\langle k\rangle }{{\langle k^{1+\theta }\rangle }^{2}}\frac{\bar{N}}{\mu }{\left(k_{i}k_{j}\right)}^{\theta }\alpha (R_{i})}] \end{array}$$ (7)

where the summation is computed over all neighboring nodes with *R*_*j*_ ≥ 1. This equation can be used to directly compute the superspreader capacity for each node in a given network. For example, Fig 3A shows a plot of superspreader capacity for a 10,000 node Erdős–Rényi (random) network, where *β* is randomly, independently assigned as a Weibull distribution with shape factor 1.2 and scale factor 2, and *μ* is equal to 1. Other parameters are $\bar{N}=1000,\theta =0.5,p=0.1$, and *μ* = 1. These data closely resemble the analytical prediction in Fig 2. A high degree of connectivity or a high *R* is sufficient for a superspreader node, although this correlation is diminished with high *R*. As expected, superspreader capacity is highly correlated with the quantity *k*^1+*θ*^ * *α*(*R*), with a linear model fit of *R*^2^ = 0.72.

In a Barabási–Albert (scale-free) model, on the other hand, only the hub nodes near the center of the network have the potential to be superspreaders. For individual nodes, a high degree of connectivity and any value of *R* greater than 1 are necessary conditions for being a superspreader. The correlation between superspreader capacity and the quantity *k*^1+*θ*^ * *α*(*R*) is *R*^2^ = 0.88, but this is mostly because nodes with high degree also tend to have a high superspreader capacity. Among high degree nodes, there is a strong positive correlation between *R* and superspreader capacity, but this correlation is not apparent among lower degree nodes.

In the Watts-Strogatz (small-world) model, every node with *R* > 1 is equally likely to be a superspreader, regardless of its degree of connectivity. This network has the opposite properties of the scale-free network, in that there is a strong positive correlation between *R* and superspreader capacity, in terms of *α*(*R*) (*R*^2^ = 0.84), but a weak correlation between degree of connectivity and superspreader capacity (*R*^2^ = 0.11). This is due to a small-world model having about four times as many edges as a scale-free model, so it is much more likely that any given node is connected to other nodes with high *R*.

### Time-dependent superspreader capacity

To determine a formula for time-dependent superspreader capacity, we focus on a single node and its neighbors, rather than the network as a whole. Consider a city that is connected to *k* other cities by road. At time *t* = 0, a number of people *I*_0_ in the hub city have been infected with a contagious illness. Assume the infection spreads exponentially, a valid assumption in the early stages of the outbreak when nearly the entire population is susceptible. The number of infected people over time is therefore given by

$$\begin{array}{c} I\left(t\right)=I_{0}e^{(\beta -\mu )t} \end{array}$$ (8)

where *β* and *μ* are the infection and recovery rates, respectively [31].

The probability that a given individual leaves the hub city for one of the neighboring cities between times *t* and *t* + d*t* is given by *p* * d*t*, where *p* is the diffusion rate. The probability that individual moves to a particular neighboring city is given by *κ*_1_, *κ*_2_, etc. Note that *κ*_1_ + *κ*_2_ + ⋯ + *κ*_*k*_ = 1, and that they are constant among all individuals and over time. In a homogeneous system, *κ*_*i*_ = 1/*k* for all *i*, and in a traffic-dependent system, $\kappa_{i}=\frac{k_{i}^{\theta }}{\sum k_{i}^{\theta }}$. Assume each individual’s movement is independent from each other.

Therefore, the probability that an individual moves from the hub city to neighboring city *i* between times *t* and *t* + d*t* is given by

$$\begin{array}{c} p\kappa_{i}dt \end{array}$$ (9)

and the probability that at least one infected individual moves from the hub city to city *i* is given by

$$\begin{array}{c} 1-{\left[1-p\kappa_{i}dt\right]}^{I(t)}≃I\left(t\right)p\kappa_{i}dt \end{array}$$ (10)

Therefore, *f*_*i*_(*t*) = *I*(*t*)*pκ*_*i*_ is the probability density function for the event that at least one infected individual moves from the hub city to city *i* at time *t*.

The probability that at least one infected individual travels between the hub city and city *i* between time 0 and time *T* is given by

$$\begin{array}{c} F_{i}\left(T\right)=\int_{0}^{T}\;I\left(t\right)p\kappa_{i}\;dt=\int_{0}^{T}\;I_{0}e^{(\beta -\mu )t}p\kappa_{i}\;dt=p\kappa_{i}\frac{I_{0}}{\beta -\mu }(e^{(\beta -\mu )T}-1) \end{array}$$ (11)

This probability is equal to 0 at time 0 and increases to 1 at time $T_{1}=\frac{1}{\beta -\mu }\;\text{log}\;[1+\frac{\beta -\mu }{pI_{0}\kappa_{i}}]$.

The expected value of the time until the first infected individual travels from the hub city to city *i* is given by

$$\begin{array}{c} \begin{array}{ccc} E_{i} & = & \int_{0}^{\infty }\;\left[1-F_{i}\left(T\right)\right]\;dT\\ & = & \int_{0}^{T_{1}}\;[1-p\kappa_{i}\frac{I_{0}}{\beta -\mu }(e^{(\beta -\mu )T}-1)]\;dT\\ & = & -\frac{1}{\beta -\mu }+[\frac{p\kappa_{i}I_{0}+\beta -\mu }{{\left(\beta -\mu \right)}^{2}}]\;\text{log}\;[1+\frac{\beta -\mu }{pI_{0}\kappa_{i}}] \end{array} \end{array}$$ (12)

The probability that at least one infected individual moves from the hub city to *at least* one neighboring city between time 0 and time *T* is given by

$$\begin{array}{c} \begin{array}{ccc} F_{any}\left(T\right) & = & 1-\prod_{i=1}^{k}\left[1-F_{i}\left(T\right)\right]\\ & = & 1-\prod_{i=1}^{k}[1-p\kappa_{i}\frac{I_{0}}{\beta -\mu }(e^{(\beta -\mu )T}-1)] \end{array} \end{array}$$ (13)

We evaluate this product with a binomial expansion and ignore higher order terms:

$$\begin{array}{c} \begin{array}{ccc} F_{any}\left(T\right) & ≃ & 1-[1-(\sum_{i=1}^{k}\kappa_{i})p\frac{I_{0}}{\beta -\mu }(e^{(\beta -\mu )T}-1)]\\ & = & p\frac{I_{0}}{\beta -\mu }(e^{(\beta -\mu )T}-1) \end{array} \end{array}$$ (14)

This probability approaches 1 at time $T_{2}=\frac{1}{\beta -\mu }\;\text{log}\;[1+\frac{\beta -\mu }{pI_{0}}]$.

Then the expected value of the time until the first infected individual leaves the hub city to any neighboring city is given by

$$\begin{array}{c} \begin{array}{ccc} E_{any} & = & \int_{0}^{\infty }\;\left[1-F_{any}\left(T\right)\right]\;dT\\ & = & \int_{0}^{T_{2}}\;[1-p\frac{I_{0}}{\beta -\mu }(e^{(\beta -\mu )T}-1)]\;dT\\ & = & -\frac{1}{\beta -\mu }+[\frac{pI_{0}+\beta -\mu }{{\left(\beta -\mu \right)}^{2}}]\;\text{log}\;[1+\frac{\beta -\mu }{pI_{0}}] \end{array} \end{array}$$ (15)

which is incidentally the same value as *E*_*i*_ but with *κ*_*i*_ replaced with 1.

The probability that at least one infected individual leaves the hub city to each of the neighboring cities is given by

$$\begin{array}{c} \begin{array}{ccc} F_{all}\left(T\right) & = & \prod_{i=1}^{k}F_{i}\left(T\right)\\ & = & \prod_{i=1}^{k}p\kappa_{i}\frac{I_{0}}{\beta -\mu }(e^{(\beta -\mu )T}-1)\\ & = & [p\frac{I_{0}}{\beta -\mu }(e^{(\beta -\mu )T}-1){]}^{k}*\prod_{i=1}^{k}\kappa_{i} \end{array} \end{array}$$ (16)

Unfortunately, we cannot assume that *T* is sufficiently small to approximate the exponential term as a polynomial. This probability approaches 1 at $T_{3}=\frac{1}{\beta -\mu }\;\text{log}\;[1+\frac{\beta -\mu }{pI_{0}}(\prod_{i=1}^{k}\kappa_{i}{)}^{-1/k}]$.

Finally, the expected value of the time it takes for an infected individual to travel from the hub city to all neighboring cities is given by

$$\begin{array}{c} \begin{array}{ccc} E_{all} & = & \int_{0}^{\infty }\;\left[1-F_{all}\left(T\right)\right]\;dT\\ & = & \int_{0}^{T_{3}}\;[1-[p\frac{I_{0}}{\beta -\mu }(e^{(\beta -\mu )T}-1){]}^{k}*\prod_{i=1}^{k}\kappa_{i}]\;dT \end{array} \end{array}$$ (17)

This function is not analytically solvable, but it can be computed numerically for given values of *p*, *I*_0_, *β*, *μ*, *k*, and *κ*_*i*_. The value of *β* will always be greater than *μ*, as a requirement for an outbreak scenario to occur, and we can consider the two extreme cases for *β*. In the case that *β* ≃ *μ*, *E*_*all*_ can be approximated as:

$$\begin{array}{c} \begin{array}{ccc} E_{all} & ≃ & \int_{0}^{T_{3}}\;[1-{\left(pI_{0}T\right)}^{k}*\prod_{i=1}^{k}\kappa_{i}]\;dT\\ & = & \frac{1}{pI_{0}}\frac{k}{k+1}{\left(\prod_{i=1}^{k}\kappa_{i}\right)}^{-1/k} \end{array} \end{array}$$ (18)

In the case that *β* ≫ *μ*, *E*_*all*_ can be approximated as:

$$\begin{array}{c} \begin{array}{ccc} E_{all} & ≃ & \int_{0}^{T_{3}}\;[1-[p\frac{I_{0}}{\beta -\mu }e^{(\beta -\mu )T}{]}^{k}*\prod_{i=1}^{k}\kappa_{i}]\;dT\\ & = & \int_{0}^{T_{3}}\;[1-[p\frac{I_{0}}{\beta -\mu }{]}^{k}e^{k(\beta -\mu )T}*\prod_{i=1}^{k}\kappa_{i}]\;dT\\ & ≃ & \frac{1}{k(\beta -\mu )}[k\;\text{log}\;[\frac{\beta -\mu }{pI_{0}}(\prod_{i=1}^{k}\kappa_{i}{)}^{-1/k}]-1] \end{array} \end{array}$$ (19)

As another note, if human movement is homogeneous and *κ*_1_ = *κ*_2_ = ⋯ = *κ*_*k*_, then *κ*_*i*_ = 1/*k* for all *i* and ${\left(\prod_{i=1}^{k}\kappa_{i}\right)}^{-1/k}$ is equal to *k*.

We can therefore define a time-dependent superspreader capacity as the velocity at which a node spreads the disease to at least one neighboring node:

$$\begin{array}{c} V_{any}=\frac{1}{E_{any}}=\{-\frac{1}{\beta -\mu }+[\frac{pI_{0}+\beta -\mu }{{\left(\beta -\mu \right)}^{2}}]\;\text{log}\;[1+\frac{\beta -\mu }{pI_{0}}]{\}}^{-1} \end{array}$$ (20)

or the velocity at which the node spreads the disease to each of its neighbors:

$$\begin{array}{c} V_{all}=\frac{k}{E_{all}}≃\frac{k^{2}\left(\beta -\mu \right)}{k\;\text{log}\;[\frac{\beta -\mu }{pI_{0}}(\prod_{i=1}^{k}\kappa_{i}{)}^{-1/k}]-1} \end{array}$$ (21)

**Fig 4:**
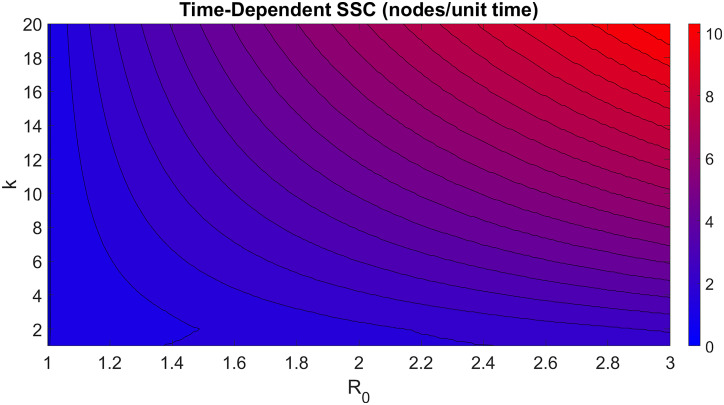
Estimation of time-dependent superspreader capacity for a node in an uncorrelated network graph. Superspreader capacity is defined as the expected velocity at which a node will spread the outbreak to all its neighbors with *R* ≥ 1. We do not expect the structure of the network to affect this relationship significantly.

A contour plot of *V*_*all*_ is shown in Fig 4, with *μ* = 1, *R* = *β*/*μ* = *β*, *p* = 0.5, *I*_0_ = 1, and assuming homogenous movement. Similar to the computed results of probability-dependent superspreader capacity, time-dependent superspreader capacity increases both with *R* and *k*. However, time-dependent superspreader capacity increases indefinitely with *R*, without diminishing returns, and its value is not dependent on the *R* value of the node’s neighbors. Because of the $(\prod_{i=1}^{k}\kappa_{i}{)}^{-1/k}$ term in the denominator of *V*_*all*_, we expect the superspreader capacity of networks with more equitable distributions of nodal degree, such as small-world networks, to be higher than in networks characterized by hub nodes such as scale-free networks. Unlike probability-dependent superspreader capacity, we do not expect time-dependent superspreader capacity to vary significantly across generations.

### Numerical simulations of superspreader capacity

An analytical model is insufficient to describe the relationship between more abstract network properties, such as centrality and clustering, to superspreader capacity. Therefore, we supplement our model with a Monte Carlo numerical simulation. As described previously, to test the hypothesis that degree of connectivity and *R* each increase superspreader risk, we randomly generated 500 metapopulation networks, each with about 500 nodes, and each network was simulated 20 times with the initial disease cases occurring in a different node. For each network, the mean values of probability-dependent and time-dependent superspreader capacity were linearly correlated with a correlation coefficient of 97.8%. Therefore, the two metrics of superspreader capacity have very similar means, but different variances. Depending on the point of origin, probability-dependent superspreader capacity varied by about 28%, and time-dependent superspreader capacity varied by about 16%. Running more than 20 simulations did not significantly reduce these variances. When modeling risk indices for future epidemics, time-dependent superspreader capacity may be a more reliable metric if the origin point of the epidemic is unknown.

The output of the Monte Carlo simulation was a list of about 250,000 nodes, about 36% of which had a non-zero time-dependent superspreader capacity, meaning they spread the epidemic to at least one neighboring node. Each node was assigned a risk index, ranging from 0 to 1, based on its superspreader capacity relative to the other nodes within the same network.

**Fig 5:**
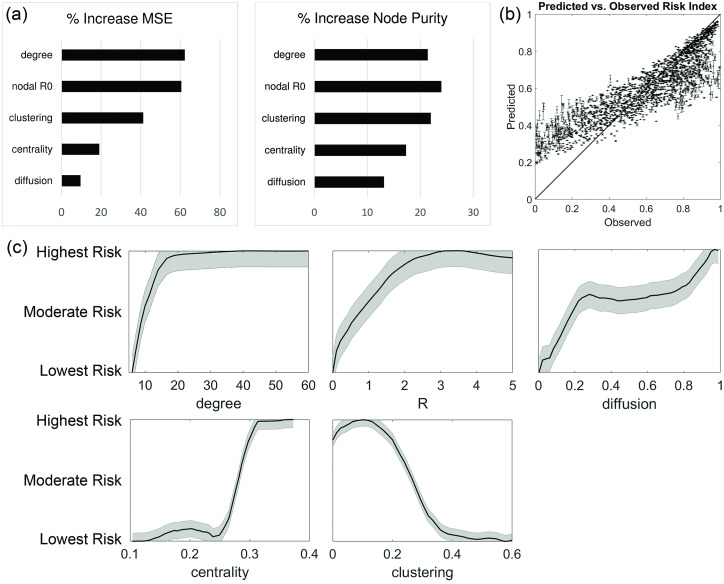
One-variable response curves of superspreader risk in terms of degree, *R*, diffusion, centrality, and clustering. Note that these curves have been rescaled for clarity. A scatter plot showing observed versus predicted risk indices for individual nodes is also shown, with a line indicating a 1:1 relationship.

We ran a random forest model on 2000 randomly selected training nodes with degrees of connectivity equal to five or greater. After five hundred iterations, the mean square residual error among training nodes was 6.1%. The model revealed that the most important network and epidemic properties for determining the risk index of an individual node, in descending level of importance, are degree of connectivity, *R*, clustering, centrality, and diffusion. Clustering coefficient is negatively correlated with risk index, the other parameters are all positively correlated. Fig 5 shows the normalized response curve of superspreader risk in terms of each of these factors. Note that for the sake of clarity, each curve has been rescaled to range from “Lowest risk” to “Highest risk.” Fig 5 also shows a scatter plot of observed versus predicted risk factor for a randomly selected set of 2000 testing nodes. The root mean squared error in prediction accuracy was 16.5%. This statistical model can predict the presence of nodes in the 90% percentile of risk index with about a 75% success rate, with a Type I error rate of 23% and a Type II error rate of about 25%. In contrast, a model that considers only degree of connectivity or only *R* has about a 45% success rate (not shown).

**Fig 6:**
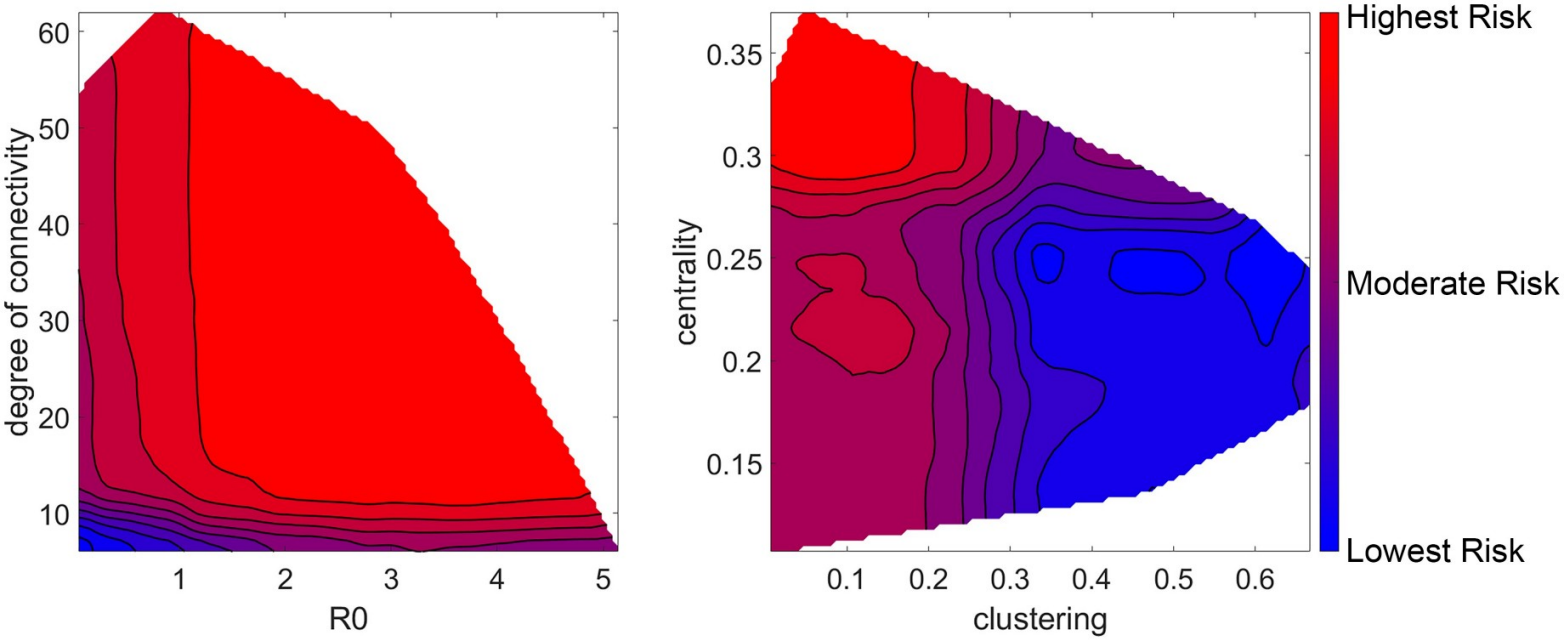
Two-dimensional response curves of superspreader risk index. Each contour line represents one decile of risk, and each shaded area contains roughly 10% of all tested nodes.

Fig 6 shows the two-dimensional response curves of superspreader risk in terms of *R* and degree of connectivity on the left, and clustering and centrality on the right. Note that the contours are colored by decile, that is, each shaded region represents 10% of tested nodes. For example, the highest risk region on the *R*-degree of connectivity chart takes up the largest area on the chart, but because degree of connectivity is power-law distributed and *R* is exponentially distributed, a node only has a 10% chance of lying within that area. These graphs show that a high degree of connectivity is a sufficient condition to be a moderate-risk node, but both high connectivity and high *R* are necessary for a high-risk node, although connectivity plays a greater role. Similarly, low clustering is a sufficient condition to be a moderate-risk node, but low clustering and high centrality are necessary for a high-risk node.

## Discussion

A critical task in epidemic preparedness is to conduct place-based health interventions for population centers that face the highest risk of becoming superspreaders. While previous research has considered the structure of the metapopulation network or spatial heterogeneity in infection rates as predictors of superspreader potential, limited research has considered both factors simultaneously. This manuscript improves on previous knowledge by considering analytically and numerically how both of these factors contribute to nodal risk of superspreader events. Our analyses show that nodal degree of connectivity is indeed the most important factor in determining superspreader capacity, consistent with the mechanistic results demonstrated in [31]. However, the value of *R*, which may be spatially heterogeneous, is the second most important, followed by other network characteristics such as clustering, centrality, and diffusion. This research extends previous research that demonstrated the superspreader capacity of nodes with high connectivity and centrality [9, 12, 32] and the importance of infection hot-spots to the spread of disease outbreaks [33–35]. This article also complements research that discuss heterogeneous infection rates among subpopulations within a group [36].

The numerical model of risk conforms well with the analytical models presented in the Results section. Degree of connectivity is strongly correlated with superspreader risk only up to about *k* = 15, after which the risk index increases only slightly with degree. However, note the difference between the likelihood and severity of a superspreader event. The probability of a particular node becoming a superspreader does not increase indefinitely with degree of connectivity, but the velocity at which that node could potentially spread the disease does increase indefinitely. This implies that a different calculus must be made for identifying superspreader nodes and estimating the potential harm caused by those nodes. For a scale-free network in particular, the node at the center of the network is almost always the highest risk superspreader, unless its *R* value is less than 1. This analysis also implies that any mitigation strategy that reduces a node’s degree of connectivity, such as travel restrictions, is almost always worthwhile to reduce the rate at which individuals become infected, but extreme mitigation might be necessary to reduce the total number of individuals that become infected over the course of the epidemic.

Although less important than connectivity, the relationship between node centrality and superspreader risk also is positively correlated. Most of the increase in risk occurs when centrality is greater than 0.25, which encompasses about 10% of all nodes in our simulations. In other words, a given node is likely to have a low risk index if within the bottom 90% of nodes, in terms of centrality, but a moderate to high-risk index if within the top 10% of nodes. As a point of comparison, there are 3,143 incorporated counties in the United States, and based on highway and air routes, approximately 300 counties would be high-risk superspreaders based on this metric [37, 38]. The inverse relationship between clustering and superspreader risk is less intuitive. Essentially, nodes with high clustering coefficients have several neighboring nodes that are also adjacent to each other, so the spreading disease is not forced to spread through the node under consideration. Although networks with high rates of diffusion are more prone to spreading the epidemic over shorter time scales [39], diffusion does not significantly affect the prevalence of individual superspreader nodes. This analysis does not consider the presence of community structures, such as a group of nodes that are densely clustered with each other but are loosely connected to neighboring nodes [40, 41]. The effect of nodal communities on superspreader risk merits further research. In addition, future analysis should consider the case in which movement between adjacent nodes is not symmetric, which would require a directed network graph structure for the metapopulation network, as well as networks with multiple layers of mobility [42].

The relationship between *R* and risk strongly resembles the *α*(*R*) function in Eq (2), in that risk increases with *R* but converges at about *R* = 3. For the small number of nodes with *R* much greater than 3, there was no correlation between their *R* values and the area or velocity of the epidemic spread originating from those nodes. This is consistent with case studies in vector-borne and directly transmitted diseases, as an *R* value of 3 is typically considered the threshold for a superspreader event [43–45]. Spatial heterogeneity in *R* can take several forms, each of which has different implications for evaluating superspreader risk. The most common form is continuously varying *R*, which typically applies when infection risk is correlated with climate or environmental factors [46–48]. In these cases, *R* does not tend to differ significantly between adjacent nodes, and superspreader nodes are likely to be concentrated in one section of the network. The second form consists of a few hotspot nodes of high *R*, surrounded by a network with low to moderate *R*. This is relevant to diseases that are prevalent in high-population centers [49], and these locales tend to be particularly effective superspreaders relative to their neighbors. The third and fourth forms, which are not addressed in this paper, are cases in which *R* can vary over time, usually as a response to changes in policy during an ongoing epidemic [50, 51], and cases in which *R* can vary within a node, such as diseases that have varying *R* values among different species [52]. Both these cases require alternative methods of measuring superspreader capacity than what is considered in this article.

An unanticipated result of the random forest model for superspreader risk is that there is some small degree of risk in nodes with high connectivity and *R* < 1, whereas the analytical models assume that these nodes have no superspreader risk at all. A moderately high value of *R* or degree of connectivity is a sufficient condition for a node to pose a superspreader risk, and if the node has a degree higher than 10, it is not necessary for *R* to be greater than 1 for the node to be a superspreader by this method of measurement. The literature does not typically consider such nodes to be outbreak sites, let alone candidates to become superspreaders, and what is likely occurring is that a high number of infected individuals are passing through the node in question, rather than originating from that node. A city that contains a highly trafficked airport but is itself a poor environment for reproduction of the pathogen might be mistaken for a superspreader if the true origins of infected individuals expanding from that node are not considered [5]. This does imply that in a city with high degree of connectivity but low *R*, restricting travel only from other high risk locations may be sufficient mitigation to reduce spreading.

Previous research has extensively examined either network factors or *R*_0_ heterogeneity as a mechanistic pathway to superspreading events, while very few efforts have attempted to combine them. Our study combines both with a particular focus on the network and habitat suitability properties of individual nodes, as opposed to more complex models that holistically consider the local and global network structure surrounding each node [10–13]. The random forest model tends to overestimate the risk index of low-risk nodes, and underestimate the risk index of high-risk nodes, by an average of 10%. The precision of the model could likely be improved by considering properties such as meta-centrality in conjunction with *R* and would be relatively straightforward to implement, at the potential cost of narrowing the applicability of the model. The advantage of the model developed in this paper is that the mechanisms that drive superspreader capacity are easily interpreted, and therefore can be applied to a wide variety of epidemic case studies. For example, the relevant balance of superspreader risk factors depends on the nature of the infectious disease in question. Malaria, tends to be more prevalent in rural environments [53], so population centers may have an inverse correlation between degree of connectivity and *R*, and there may be fewer nodes in the high-risk threshold. Directly transmitted diseases such as influenza and COVID-19, on the other hand, tend to demonstrate a positive correlation between *R* and population density [54]. In this case, the distribution of superspreader capacity among nodes may be more bimodal, as the highly connected nodes will have extremely high superspreader capacity, and vice versa. For severely contagious diseases with *R*_0_ > 2 throughout the entire network, small differences in *R* may have no bearing on risk when compared to network connectivity. This method of predicting superspreaders works equally well with other epidemic models, such as Susceptible-Infected-Susceptible (SIS), because the superspreader event typically occurs on a faster time scale than recovery or reinfection.

In addition to predicting superspreader risks within a metapopulation network, the model discussed in this paper may help predict the relative effectiveness of various epidemic mitigation strategies [51, 55]. For example, closing airports or blocking highways has the effect of reducing a node’s degree of connectivity and centrality, although the effectiveness of these methods is under question [56], and they require substantial social data to develop an accurate mobility model [57]. Improved hygienic practices and medical resources may reduce the effective *R* value in regions that follow these practices, which may reduce superspreader risk if *R* can be reduced to less than 3 [58, 59]. This model can also be used to predict the effectiveness of mitigation strategies if they are not applied uniformly across the network; for example, if only a subset of counties enforce social distancing guidelines to reduce the spread of a directly transmitted pathogen [60]. Future research should also take into consideration nonhomogeneous distributions of population density. Within a given population center, it must be taken into consideration that only a certain fraction of its population will engage in proper mitigation strategies to prevent an epidemic outbreak, which would contribute to a variable *R* within a node. It is also necessary to test how the shut-down of certain community hubs, such as a school or a shopping center, affects the superspreader risk of the entire network. This analysis may require a more advanced algorithm to generate random metapopulation networks based on multiple spatial scales [25].

## Conclusion

This study demonstrates how spatially heterogeneous disease reproduction rates can affect the superspreader potential of nodes across a human metapopulation network. The statistical model, based on a random forest algorithm, could be deployed to predict the risk indices of epidemics and analyze the relative effectiveness of containment and mitigation strategies. The model could be improved by considering other network properties of the network, or the superspreader capacity of neighboring nodes evaluated recursively, but this model strikes a balance between effectiveness and simplicity. A key finding of this study is that the risk of a certain population node becoming a superspreader increases convergently with *R* and with degree of connectivity. A value of *R* or degree of connectivity individually above a certain threshold are sufficient for a node to be a moderate-risk superspreader, but both are necessary to be a high-risk superspreader. This finding suggests a balanced approach to addressing both *R*_0_ and network connectivity to achieve optimal epidemic management scenarios.
